# Gut microbiota signature in children with autism spectrum disorder who suffered from chronic gastrointestinal symptoms

**DOI:** 10.1186/s12887-023-04292-8

**Published:** 2023-09-20

**Authors:** Hui Wang, Shu Liu, Liqing Xie, Jinhui Wang

**Affiliations:** 1 Child Healthcare Department, Xiamen Children’s Hospital, Children’s Hospital of Fudan University at Xiamen, Xiamen, 361006 China; 2 Department of Clinical Laboratory, Xiamen Children’s Hospital, Children’s Hospital of Fudan University at Xiamen, No.92-98 Yibin Road, Huli District, Xiamen, 361006 China

**Keywords:** Gut microbiota, Autism spectrum disorder, Children, Metabolism

## Abstract

**Background:**

Children diagnosed with autism spectrum disorder (ASD) frequently suffer from persistent gastrointestinal symptoms, such as constipation and diarrhea. Various studies have highlighted differences in gut microbiota composition between individuals with ASD and healthy controls of similar ages. However, it’s essential to recognize that these disparities may be influenced by cultural practices, dietary habits, and environmental factors.

**Methods:**

In this study, we collected fecal samples from both children diagnosed with ASD (n = 42) and healthy individuals (n = 41) residing in the southeastern coastal region of China. Subsequently, 16 S rRNA gene sequencing and advanced bioinformatics analyses were conducted to investigate the distinctive features of gut microbial communities within each group.

**Results:**

The ASD group consisted of 28 males and 14 females, with a median age of 5.8 years, while the control group included 25 males and 16 females, with a median age of 6.8 years. Among the 83 sequenced fecal samples, a total of 1031 operational taxonomic units (OTUs) were identified. These included 122 unique OTUs specific to the control group and 285 unique OTUs specific to the ASD group. Analyses of α-diversity and β-diversity unveiled significant differences in the abundance and composition of gut microbiota between the two groups. It was found that the dominant bacterial taxa in healthy individuals were *UBA1819*, *Flavonifractor*, and *Bradyrhizobium*. In contrast, the ASD group exhibited a prevalence of *Streptococcus*, *Ruminococcus*, and *Ruminiclostridium*. Further analysis using Kyoto Encyclopedia of Genes and Genomes (KEGG) and Clusters of Orthologous Groups (COG) showed significant differences in the metabolic functionalities of the gut microbiota between the two groups. Notably, the metabolic pathway related to alpha-linolenic acid (ALA) in the gut microbiota of the ASD group was notably diminished compared to the control group. Conversely, the ASD group demonstrated significantly elevated levels of metabolic pathways involving uncharacterized conserved proteins, aminoglycoside phosphotransferase, and inorganic pyrophosphatase compared to the control group.

**Conclusions:**

Overall, these results confirm that there are significant differences in the gut microbiota structure between children with ASD and healthy controls in the southeast coastal region of China. This underscores the critical significance of delving into clinical interventions capable of mitigating the gastrointestinal and psychological symptoms encountered by children with ASD. A particularly encouraging path for such interventions lies in the realm of fecal microbiota transplantation, a prospect that merits deeper inquiry.

**Supplementary Information:**

The online version contains supplementary material available at 10.1186/s12887-023-04292-8.

## Background

Autism spectrum disorder (ASD) stands as a neurodevelopmental disorder characterized by intricate hurdles in social communication and interaction, alongside a display of constrained interests and repetitive behaviors [1, 2]. Under the umbrella term of autism spectrum disorder lie several closely linked conditions, encompassing Asperger’s syndrome, pervasive developmental disorders not otherwise specified (PDD-NOS), and childhood disintegrative disorder [3–5]. According to present epidemiological investigations, the estimated prevalence rate of ASD hovers around 1 in 100 children; however, certain regions within the United States report significantly higher rates, reaching as steep as 1 in 44 [6]. This variation in prevalence rates might be attributed to differing diagnostic criteria, variances in healthcare accessibility, and disparities in ASD awareness across specific regions. Amidst this fluctuation, the undeniable reality emerges that ASD presents a substantial public health apprehension demanding ongoing investigation and focus. Through an in-depth grasp of the factors underpinning ASD prevalence, we can more effectively recognize and bolster individuals bearing ASD and their families, while simultaneously forging potent prevention and intervention strategies. Notably, in China, the count of children affected by ASD ascends into the millions, and this figure persists in its upward trajectory. These children routinely encounter obstacles in communication, language acquisition, self-care, and the pursuit of knowledge. Furthermore, they may incline toward solitary behaviors. In severe instances, the potential exists for them to exhibit self-injurious or aggressive tendencies [7].

The etiology of ASD remains to be comprehensively deciphered. A myriad of elements, encompassing genetics, prenatal and perinatal circumstances, environmental impacts, social determinants, and family interplay, are all conceivably implicated [7]. Despite the array of interventions accessible for ASD, encompassing rehabilitative training, psychological therapy, and medication, these methods frequently prove insufficient in attaining the sought-after curative results. These constraints underscore the necessity for extended research endeavors and pioneering approaches in treating ASD [4, 8].

The human gastrointestinal tract harbors a diverse and extensive microbiota. With a population ranging from 10^13^ to 10^14^ microorganisms, the gut microbiota possesses a genome over 100 times larger than of humans [9], playing pivotal roles in the host’s well-being. It ferments indigestible food components into absorbable metabolites, synthesizes essential vitamins, eliminates harmful toxins, resists pathogens, bolsters the intestinal barrier, and regulates the immune system [10]. Extensive research underscores the crucial engagement of the gut microbiota in both the development and operation of the host’s adaptive immune system, as well as in shaping the central nervous system [11–13]. Therefore, gut microbiota dysbiosis can lead to various disorders affecting the gastrointestinal, immune, and neurodevelopmental systems, including ASD [14]. Clinical observations reveal that children with ASD frequently experience chronic gastrointestinal issues like constipation and diarrhea, with constipation being the most frequently noted [15]. Importantly, the severity of gastrointestinal symptoms in ASD-affected children correlates directly with the extent of their condition. Those grappling with persistent gastrointestinal problems tend to exhibit deficient social skills and heightened difficulty in emotional regulation. Encouragingly, studies demonstrate that enhancements in these gastrointestinal symptoms correspond to diminished severity of social and emotional difficulties in children with ASD [16].

While numerous studies have underscored distinctions in gut microbiota between individuals with ASD and their healthy counterparts, the quest for advantageous bacteria or viable metabolic products for clinically treating ASD remains unfruitful. This dearth of discovery could stem partly from the gut microbiota’s limited contribution to the onset and progression of ASD. Besides, disparities in culture, diet, environment, and customs across global regions and populations can substantially influence gut microbiota variations among individuals. Therefore, by broadening the spectrum of screened individuals, the probability of identifying microbiota associated with ASD is heightened. In this study, we aimed to analyze the gut microbiota composition by collecting fecal samples from both healthy individuals and those with ASD in the southeastern coastal region of China. Through 16 S rRNA gene sequencing, we strive to furnish an intricate depiction of gut microbiota profiles in individuals with ASD and potentially uncover novel microbiota associated with this condition.

## Materials and methods

### Subjects

We recruited a total of 42 participants with ASD, aged 2 to 8 years, from Xiamen Children’s Hospital, Children’s Hospital of Fudan University in Xiamen (China). To serve as a control group, we collected fecal samples from 41 healthy volunteers who were matched in terms of age and gender. The diagnosis of ASD was determined by two experienced child neuropsychiatrists, who followed the criteria outlined in the Diagnostic and Statistical Manual of Mental Disorders (DSM-V) and the International Statistical Classification of Diseases and Related Health Problems (ICD-10). The control group for this study was comprised of developing children who did not exhibit any symptoms of ASD and had no direct exposure to individuals with ASD. To ensure the accuracy of the results, participants who had a history of nutritional supplements, special diets, or known neurological disorders were excluded from the study. Furthermore, individuals who had received antibiotic, antifungal, probiotic, or prebiotic treatments within the three months prior to sampling were also excluded (Table S1). All methods were conducted in accordance with relevant guidelines and regulations, and all experimental protocols were approved by the Ethics Committee of Children’s Hospital of Fudan University at Xiamen (approve number: 202244). Written informed consent was obtained from the parents and/or legal guardians of all participants.

### Fecal samples

In this study, we implemented a standardized collection method [17] for the enrolled cohort in order to mitigate heterogeneity resulting from operational variations. Fecal mid-segment samples were collected from the two cohorts within 30 min after defecation. To safeguard the integrity of microbial nucleic acids within these samples, approximately 200 mg of each specimen was transferred into a fecal preservation solution provided by Treatgut Biotech, China. The solution was stored at room temperature and proved to be effective in maintaining the microbial nucleic acid for at least 6 months.

### **Bacterial DNA extraction and 16 S rRNA gene sequencing**

Microbial DNA was extracted from fecal samples using the QIAamp DNA Stool Mini Kit (Qiagen, Hilden, Germany). DNA concentration and purity were quantified using the Multiskan™ GO microplate reader and DNA integrity was checked using agarose gel electrophoresis. The V3-V4 region of the bacterial 16 S rRNA gene was amplified using specific and high coverage 341 F and 806R primers (forward primer: 5′-CCTACGGGNBGCASCAG-3′ and reverse primer: 5′-GGACTACNVGGGTWTCTAAT-3′) via PCR amplification (ABI 2720 thermal cycler; Applied Biosystems, Foster City, CA, USA). The sequencing library was then constructed using Illumina. Each sample was quantified using Qubit 3.0 and pooled to ensure homogeneity. The different libraries were pooled onto the flowcell based on their effective concentration and the desired amount of sequencing data and then sequenced using the Illumina high-throughput sequencing platform. Raw data obtained from the Illumina HiSeq/MiniSeq sequencing platform were referred to as raw reads. The high-quality clean reads were generated by merging and quality control using the Flash software, followed by filtering of chimeric sequences (Chimera_check). Operational taxonomic units (OTU) clustering was performed using the QIIME software to obtain the OTU abundance of each sample. 16 S rRNA gene sequencing data are available on BioSample (https://www.ncbi.nlm.nih.gov/bioproject/PRJNA953169; BioProject ID: PRJNA953169).

### Bioinformatics analysis

All sequencing data were analyzed using the R package (version 2.15.3). Rarefaction was applied to OTUs to reduce sampling heterogeneity and calculate the α- and β-diversity. Principal coordinate analysis (PCoA) was utilized to evaluate differences in bacterial composition based on weighted UniFrac distances. For high-dimensional biomarker discovery, the linear discriminant analysis effect size (LEfSe) algorithm was performed, which identifies taxa that exhibit differential abundance between two groups. Linear discriminant analysis (LDA) LEfSe was utilized to estimate the impact of each differentially abundant taxon and distinguish the most biologically different taxa. To compare data differences among multiple groups, either the Wilcoxon rank-sum test or Kruskal-Wallis test was conducted. Furthermore, the Spearman correlation was used to analyze the correlation between differentially abundant taxa and other indicators. Besides, to uncover differences in the gut microbiota metabolic pathways between the two cohorts, Kyoto Encyclopedia of Genes and Genomes (KEGG; www.kegg.jp/kegg/kegg1.html) [18–20] and Clusters of Orthologous Groups (COG) analyses were performed.

### Statistical analysis

Data were analyzed using SPSS 20.0 software (IBM Corp., Armonk, NY, USA). Student’s t-test was utilized to compare qualitative data with equal variance, while Welch’s t-test was used for qualitative data with unknown variance. In addition, the comparison of qualitative data between the ASD and control groups was conducted using the chi-square test. The results were presented as mean ± standard deviation, and statistical significance was determined by a significance level of P < 0.05.

## Results

### Baseline characteristics of the study cohort

The study comprised 42 children diagnosed with ASD (Table 1), of whom 28 (66.67%) were male and 14 (33.33%) were female. The median age of the participants was 5.8 years, with a range of 2.5 to 9.6 years. The control group consisted of 41 healthy children (Table 2), with 25 (60.98%) being male and 16 (39.02%) being female. The median age of the control group was 6.8 years, with a range of 3 to 8.5 years. Among the 42 ASD patients, 6 (14.29%) had a history of long-term probiotic use, while 2 (4.76%) had only used antibiotics intermittently. Additionally, 3 (7.14%) had taken antibiotics within the past 3 months, and 7 (16.67%) had utilized other medications. Regarding therapeutic interventions, 11 (26.19%) received Applied Behavior Analysis (ABA), structured training, sensory integration, and language training, while 10 (23.81%) received Early Start Denver Model (ESDM), floor time, and sensory integration training. The remaining 21 (50%) did not receive any training. Within the group of 42 ASD patients, 18 (42.86%) received personalized one-on-one training within an institutional setting, while 10 (23.81%) underwent training at home. Some patients received mixed interventions. In terms of symptomatic presentations, 19 (45.24%) patients experienced constipation, 14 (33.33%) experienced diarrhea, 10 (23.81%) faced challenges with swallowing, 10 (23.81%) exhibited anorexia, 2 (4.76%) displayed an increased appetite, 8 (19.05%) showed diminished appetite, 18 (42.86%) demonstrated selective eating tendencies, and 3 (7.14%) were diagnosed with food allergies.

**Table 1:** Characteristics of children with ASD

Characteristics	Number of cases (%)
Age (year)	
2.5–9.6(5.8)	42 (100)
Gender	
Male	28 (66.67%)
Female	14 (33.33%)
Long-term use of probiotics	6 (14.29%)
Use of medications	
Intermittent use of antibiotics	2 (4.76%)
Antibiotic use in the past 3 months	3 (7.14%)
Use of other medications	7 (16.67%)
Therapy	
ABA, structured teaching, sensory integration, speech therapy	11 (26.19%)
ESDM, floortime, sensory integration	10 (23.81%)
No training	21(50%)
Approach of Therapy	
Institutional one-on-one therapy at an institution	18 (42.86%)
Therapy at home	10 (23.81%)
Constipation	19 (45.24%)
Diarrhea	14 (33.33%)
Dysphagia	10 (23.81%)
Anorexia	10 (23.81%)
Strong appetite	2 (4.76%)
Weak appetite	8 (19.05%)
Selective eating	18 (42.86%)
Food allergy	3 (7.14%)

**Table 2:** Characteristics of the healthy control

Characteristics	Number of cases (%) of cases(%)
Age (year)	
3.0-8.5(6.8)	41 (100%)
Gender	
Male	25 (60.98%)
Female	16 (39.02%)

### Microbial community distribution in ASD and healthy control children

**Fig. 1:**
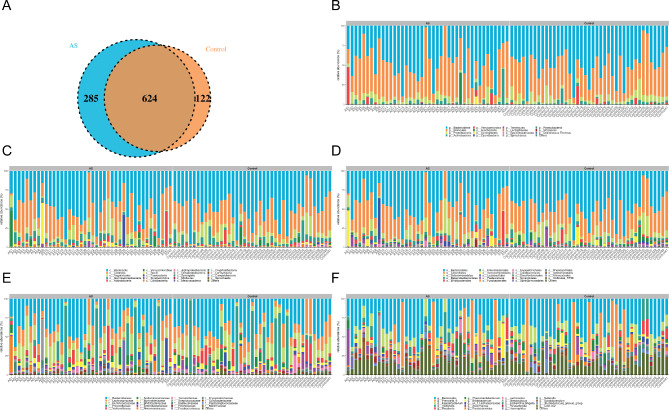
Distribution of gut microbiota in children with ASD and healthy controls. **(A)** Venn diagram of OTUs of gut microbiota in children with ASD and healthy controls. **(B-F)** Relative abundance of the 20 most abundant **(B)** phylum, **(C)** class, **(D)** order, **(E)** family, and **(F)** genus levels in the two groups. ASD, autism spectrum disorder; OTU, operational taxonomic unit

Stool samples from 42 ASD patients and 41 healthy controls were analyzed through 16 S rRNA gene sequencing. The sequences underwent a series of meticulous steps, including data quality control, sequence splicing, filtering, and the removal of chimeras, resulting in optimal sequences. These sequences predominantly fell within the 240–260 bp range, culminating in an average of 64,337 reads per sample, with a total of 63,834 reads successfully aligned. Within the 83 sequenced stool samples, a total of 1031 OTUs were identified (Table S2). Among these, 624 OTUs were found to be shared between the control and ASD groups. The control group exhibited 122 unique OTUs, while the ASD group showcased 285 unique OTUs **(**Fig. 1A**)**. Among these samples there were over 291 different genera, 89 families, 48 orders, 23 classes, and 15 phyla (Table S*g__Ruminococcus_1*, *g__Streptococcus*). Our study further elucidates the relative abundances of bacterial communities at various taxonomic levels, including phylum, class, order, family, and genus, within each group **(**Fig. 1B-F**)**. At the phylum level, the three most prevalent OTUs in the fecal samples were identified as *Bacteroidetes*, *Firmicutes*, and *Proteobacteria* (Fig. 1B). At the genus level, the dominant bacteria genera were determined to be *Bacteroides, Prevotella_9*, and *Faecalibacterium* (Fig. 1F). The fold changes between the two groups were determined for the top 10 most abundant differential genera. The control group exhibited significantly higher relative abundances of *g_Acinetobacter*, *g_Flavonifractor*, *g_Prevotellaceae_NK3B31_group*, *g_UBA1819*compared to the ASD group. Conversely, the ASD group showed significantly higher relative abundances of *g_Ruminococcus_1*, *g_Streptococcus*, *g_Lachnospiraceae_NC2004_group*, compared to the control group (Table S8).

**Analysis of the α- and β-diversity of gut microbiota in children with ASD and healthy controls**.

**Fig. 2:**
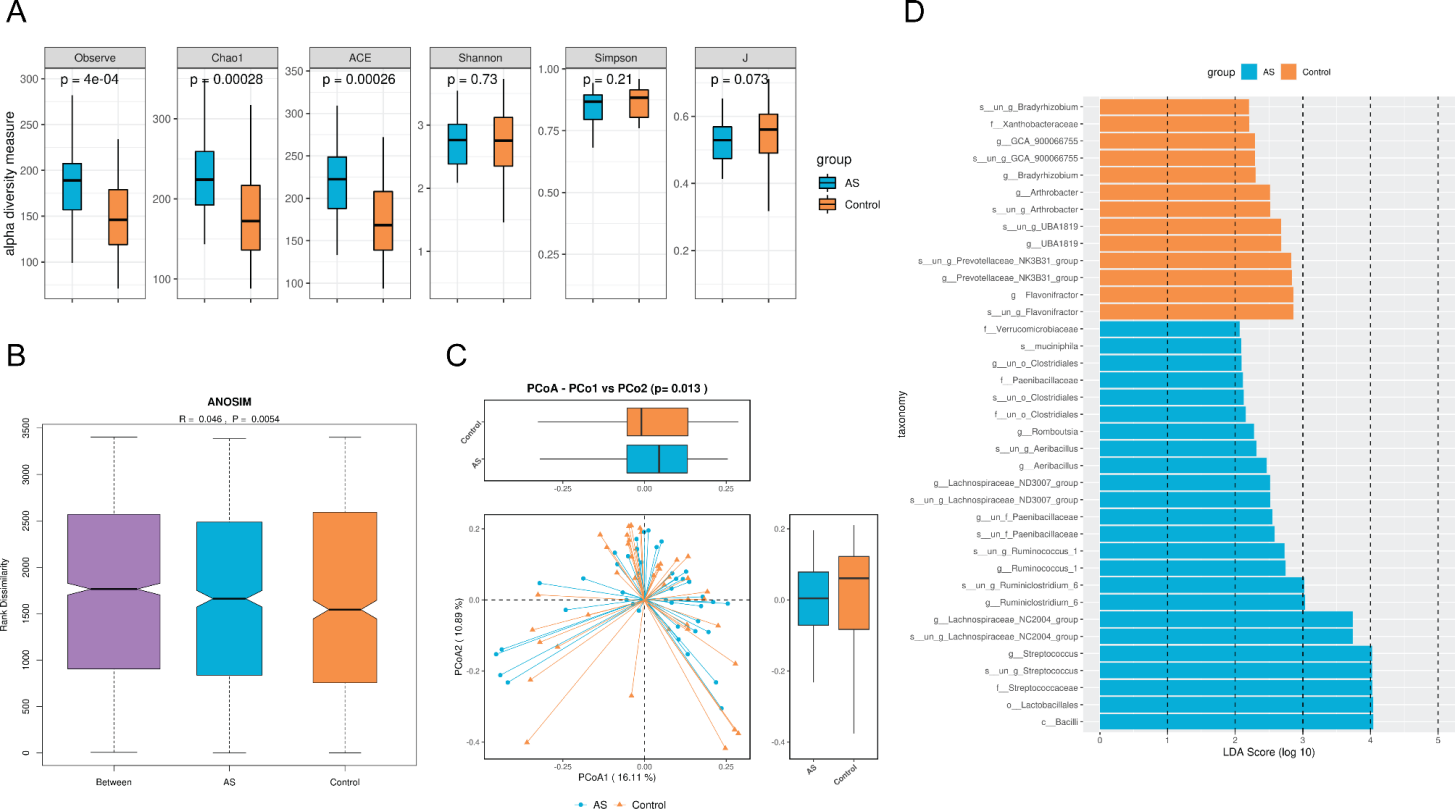
Analysis of α-diversity and β-diversity of gut microbiota in children with ASD and Healthy Controls. **(A)** Results of α-diversity of gut microbiota in children with ASD and healthy controls. **(B)** ANOSIM analysis of gut microbiota in the two groups. **(C)** PCoA analysis of gut microbiota in the two groups. **(D)** Discrimination of dominant gut microbiota between the two groups based on LDA score. ASD, autism spectrum disorder; ANOSIM, analysis of similarity; PCoA, principal coordinate analysis

We employed Shannon-Wiener curves and Simpson rarefaction curves to assess the analysis of fecal samples obtained from children with ASD and healthy controls. The smooth curves observed in both cohorts suggested satisfactory data quality and quantity, effectively capturing a substantial portion of the microbial information within the samples. The analysis of α-diversity revealed that there was no significant difference in the diversity and evenness of species present in the fecal samples of children with ASD and those of healthy controls (Shannon index, *P* = 0.69; Simpson index, *P* = 0.21; J index, *P* = 0.073) **(**Fig. 2A**)**. Nevertheless, there was a significant difference in the richness of fecal samples between the two groups, supported by the observed species index (*P* < 0.05), Chao1 index (*P* < 0.05), and ACE index (*P* < 0.05). Additionally, β-diversity analysis illuminated significant differences in gut microbiota composition between the cohorts, substantiated by a PCoA index of *P* = 0.013 **(**Fig. 2B C**)**. LDA was used to reduce the dimensionality of the data and assess the influence of significantly different species between the two groups, represented by the LDA score. The results suggested that dominant bacteria in healthy individuals included *UBA1819, Flavonifractor, and Bradyrhizobium*, whereas individuals with ASD exhibited dominant bacterial genera like *Streptococcus, Ruminococcus, and Ruminiclostridium***(**Fig. 2D**)**.

### Differences in intestinal bacterial abundance between fecal samples from children with ASD and healthy controls

**Fig. 3:**
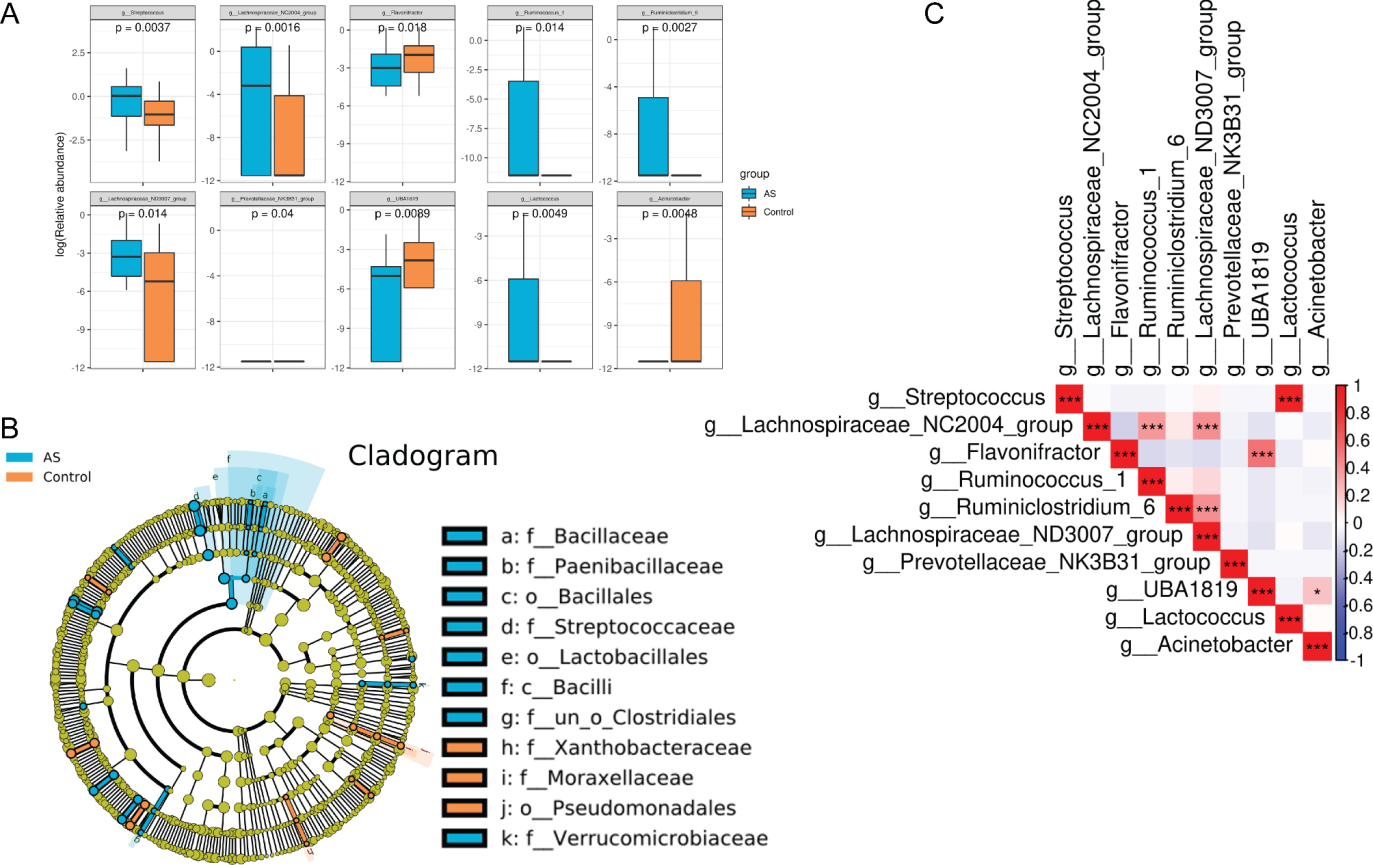
Significant differences in species abundance in fecal samples of children with ASD and healthy controls. **(A)** Bar chart of differential bacterial genera in fecal samples of children with ASD and healthy controls. **(B)** Microbial evolution analysis of gut microbiota in the two groups. **(C)** Correlation analysis of bacterial genus abundance in all samples. ASD, autism spectrum disorder

To gain further insight into the microbial composition within fecal samples from children with ASD in comparison to healthy controls, a statistical analysis of bacterial abundance was conducted. The findings showed that at the genus level, the relative abundance of bacterial genera *Streptococcus, Lachnospiraceae_NC2004, Ruminococcus, Ruminiclostridium, Lachnospiraceae_ND3007*, and *Lactococcus* in patients with ASD was significantly higher than in healthy controls (*P* < 0.05). Conversely, the relative abundance of bacterial genera *Flavonifractor, UBA1819*, and *Acinetobacter* in patients with ASD was notably lower than in healthy controls (*P* < 0.05). Further correlation analysis underscored significant positive correlation between the bacterial abundance of *Flavonifractor* and *UBA1819*, as well as between *Lactococcus* and *Streptococcus***(**Fig. 3A**)**. In addition, an analysis of microbial evolution demonstrated that there was a continuous evolutionary relationship between the gut microbiota of children with ASD at five different levels, including *Firmicutes, Bacilli, Lactobacillales, Streptococcaceae, Streptococcus, Firmicutes, Bacilli, Lactobacillales, Streptococcaceae*, and *Lactococcus***(**Fig. 3B**)**. Among them, the abundance of *Streptococcus* and *Lactococcus* displayed a significant increase in patients with ASD **(**Fig. 3C**)**. Notably, the associations between the differential bacterial taxa and clinical manifestations were investigated with Spearman’s correlation analysis (Figure S1). Childhood Autism Rating Scale (CARS) were significant negatively correlated with *g_Ruminococcus_1* and positively correlated with *g_Acinetobacter* and *g_Flavonifractor*, while none of the genera were significantly correlated with Autism Behavior Checklist (ABC).

### ASD children and healthy controls exhibit significant differences in gut microbiota metabolic pathways

**Fig. 4:**
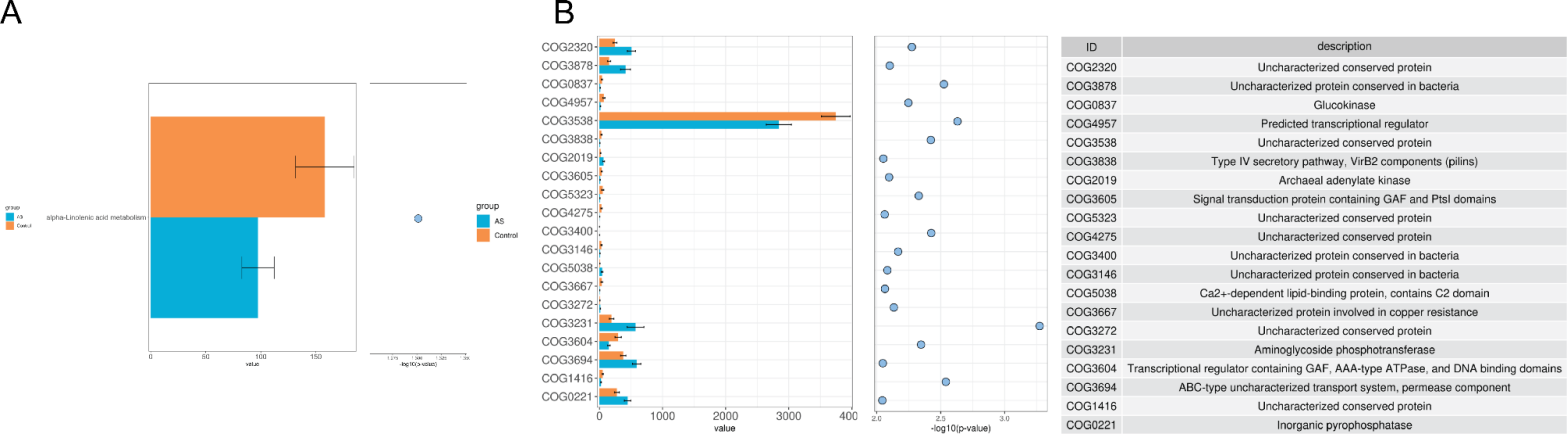
Significant differences in metabolic pathways of gut microbiota in children with ASD and healthy controls. **(A)** Analysis of metabolic pathways related to differential gut microbiota in children with ASD and healthy controls based on the KEGG database. **(B)** Analysis of metabolic pathways related to differential gut microbiota in the two groups based on the COG database. ASD, autism spectrum disorder; KEGG, Kyoto Encyclopedia of Genes and Genomes; COG, the Cluster of Orthologous Groups

Given the pivotal role that small molecule metabolites originating from the gut microbiota play in the progression of the disorder, our study focused on analyzing the metabolic pathways. Through KEGG and COG analyses, we were able to uncover noteworthy differences in the gut microbiota metabolic pathways between the two cohorts. Notably, our findings indicate a significant reduction in the alpha-linolenic acid metabolism pathway within the gut microbiota of the ASD group, in comparison to the healthy group **(**Fig. 4A**)**. Moreover, the COG analysis revealed noteworthy distinctions. Specifically, the uncharacterized conserved protein within the ASD group exhibited a substantial decrease compared to healthy controls. In contrast, the uncharacterized conserved protein, aminoglycoside phosphotransferase, and inorganic pyrophosphatase displayed notably elevated levels in comparison to those observed in healthy subjects. **(**Fig. 4B**)**.

## Discussion

The emergence of ASD during childhood is closely linked to the intricate world of gut microbiota. Nonetheless, owing to the intricate interplay of factors like culture, dietary habits, environment, and customs, substantial disparities in gut microbial communities exist among individuals inhabiting diverse geographical regions [21, 22]. Presently, there is a lack of information regarding the distribution of gut microbiota among children with ASD in the southern expanse of China. Consequently, the objective of this study is to bridge this knowledge gap by collecting fecal samples from both healthy children and those diagnosed with ASD residing in the southeastern coastal area of China, specifically Fujian Province. The 16 S rRNA gene sequencing was utilized to analyze gut microbiota in children with ASD. As a demonstrative example, Fig. 1C presents the AS30 sample, exhibiting a significantly high abundance of *c_Fusobacteria*. Likewise, Fig. 1D highlights the AS9 sample, indicating a notable increase in *o_lactobacillales*. Consistent with previous research, it was observed that children diagnosed with ASD exhibit an atypical gut microbial composition in comparison to their typically developing peers [23–25]. Certain pathogenic bacteria were found to be present at higher abundance in gut microbiota of children with ASD than in normal children, such as *Clostridia, Bacteroidetes*, and *Desulfovibrio* [26]. These results provide further evidence that gut microbial composition of children with ASD significantly differs from that of healthy children.

The taxa exhibiting significant abundance differences between the ASD and control cohorts were identified. In the control group, *g_Acinetobacter*, *g_Flavonifractor*, *g_Prevotellaceae_NK3B31_group*, and *g_UBA1819* displayed higher abundance compared to the ASD group. Conversely, the ASD group exhibited higher abundance of *g_Ruminococcus_1*, *g_Streptococcus*, and *g_Lachnospiraceae_NC2004_group* when compared to the control group. These observed changes in differential genera between the two groups align with the findings of several previous studies. For instance, Zhang et al. observed the proportion of Firmicutes/Bacteroidetes in ASD was significantly increased, accompanied by pronounced enrichment of *Lachnospiraceae* and *Ruminococcaceae* families. In their study, eighteen genera, including *Ruminococcus*, *Blautia*, and *Holdemanella* were notably elevated in the ASD cohort [27]. Additionally, Li et al. employed 16 S rRNA gene sequencing to analyze stool samples from 59 mother-child pairs with ASD children and 30 matched pairs of healthy children, revealing distinctive bacterial biomarkers like *Alcaligenaceae* and *Acinetobacter* within the ASD group [28]. These findings collectively underscore the variability of microbial community structures across different cohorts and regions, highlighting the need for diverse regional sampling to pinpoint region-specific bacteria potentially linked to disease.

Given that gut microbiota structure of children with ASD undergoes significant changes, researchers have delved into the potential of modulating gut microbiota as a therapeutic measure for this condition. In a previous study, 18 children with ASD underwent a regimen of Fecal Microbiota Transplantation (FMT) spanning 18 weeks. Following an 8-week period of FMT treatment, marked enhancements were observed in the scores of the ASD Diagnostic Observation Schedule (ADOS), the ABC, and the Social Responsiveness Scale (SRS) in children with ASD, in comparison to their pre-treatment scores. Additionally, the Gastrointestinal Symptom Rating Scale (GSRS) revealed significant improvement in gastrointestinal symptoms, including constipation, diarrhea, indigestion, and abdominal pain [15]. successful engraftment of certain donor microbial communities in the gut, leading to heightened diversity of the gut microbiota. These findings suggest the potential efficacy of FMT as a short-term intervention for alleviating both social and gastrointestinal symptoms in children with ASD. Furthermore, these outcomes hint at the possibility of FMT serving as a viable long-term approach for ASD treatment [15]. Given the variances in microbial community structures across populations and regions, accompanied by differing bacterial species accountable for these distinctions, it becomes apparent that personalized microbial FMT might represent a feasible avenue. Recent investigations have illustrated that aligning the gut microbiota of fecal donors with FMT recipients can effectively address conditions like ulcerative colitis (UC). This matching paradigm offers the prospect of selecting well-suited fecal donors for patients with UC [29, 30]. Hence, a promising direction for improving treatment efficacy in children with ASD could be applying the donor-recipient matching FMT strategy.

The clinical manifestations of childhood ASD are varied, and aside from mental symptoms, gastrointestinal symptoms also play a significant role in diagnosis [15, 16]. As previously observed, there is a noticeable alteration in the structure of intestinal microbiota in individuals with ASD; however, the precise causal relationship between the two remains incompletely comprehended. Recent studies have elucidated the crucial role of bacterial metabolites in regulating diverse facets of the host’s metabolism, including but not limited to appetite regulation, glucose homeostasis, energy expenditure, and immune response. These metabolites serve as intermediary agents through which the gut microbiota exerts its effects on the host [31, 32]. Gut microbiota dysbiosis causes significant alterations in the metabolomic profile, characterized by elevated levels of short-chain fatty acids (SCFAs) such as propionic and butyric acids. These changes can be attributed to the substantial proliferation of bacterial genera *Clostridia, Bacteroidetes*, and *Desulfovibrio*, which have been previously linked to patients with ASD [33]. Particularly noteworthy is the augmented presence of Clostridia, known producers of propionic acid, which has been observed to be significantly higher in the fecal samples of ASD children suffering from constipation. Moreover, the heightened levels of propionic acid in fecal samples align with this, suggesting a potential link between constipation and the abundance of propionic acid-producing bacteria [34, 35]. In our study, we utilized bioinformatics analysis to confirm the changes in metabolic pathways that corresponded to different microbial communities in local ASD patients. Our study revealed a significant reduction in alpha-linolenic acid metabolism, paving the way for deeper exploration into the intricate molecular mechanisms that underpin the impact of gut microbiota on the development of ASD.

Our study possesses several limitations. Firstly, our analysis focused on bacterial profiles derived from the 16 S rRNA gene. Consequently, this examination omitted the finer strain-level distinctions within gut bacterial taxa, as well as other microbial components such as the fungal microbiota and virome. Secondly, conducting fecal collection at different time frames and employing longitudinal sampling is a valuable approach to comprehensively study the gut microbiota’s dynamics, understand its responses to various factors, and uncover potential associations with health outcomes. It helps mitigate the inherent variability of the microbiota and provides a more accurate representation of its behavior over time [36, 37]. By analyzing a disease-free Swedish population over a year, utilizing both whole-genome metagenomic and 16 S rRNA gene sequencing, study by Olsson LM et al. underscores the significance of temporal dynamics in the gut microbiota, which can impede the identification of consistent microbial markers associated with health [36]. Moreover, longitudinal analysis about ASD suggests that variations in lethargy/social withdrawal levels among individuals at different time points exhibited a correlation with alterations in gut microbiome composition. Additionally, a decline in gut microbiome diversity was found to be linked to an exacerbation of inappropriate speech between the aforementioned time points [37]. The composition of the gut microbiota is not fixed and undergoes ongoing adaptive modifications. Paradoxically, He et al. demonstrated the stability of gut microbial communities within a specific timeframe [17]. Frost et al. observed a remarkable stability in the overall structure of fecal microbiome communities over time at a population level [38]. In the current analyses, we did not employ a longitudinal sampling approach to evaluate the dynamic changes in gut microbiota over time. Our assessment of the gut microbiota in ASD children was constrained to data from a single sampling time point. Hence, to gain a more comprehensive understanding of the dynamic nature of gut flora in ASD patients, we intend to establish multiple sampling time points and implement diverse intervention protocols in our future investigations.

## Conclusions

Overall, this study recruited ASD children and healthy children in Fujian, China, and collected their fecal samples for intestinal microbiota testing, confirming significant differences in the microbiota structure between the two groups. These findings offer evidence to support the development of clinical interventions that aim to alleviate gastrointestinal and mental symptoms in children with ASD.

### Electronic supplementary material

Below is the link to the electronic supplementary material.

Supplementary Material 1

Supplementary Material 2

Supplementary Material 3

Supplementary Material 4

Supplementary Material 5

Supplementary Material 6

Supplementary Material 7

Supplementary Material 8

Supplementary Material 9

Supplementary Material 10

## Data Availability

In this study, 16 S rRNA gene sequencing data are available on BioSample (https://www.ncbi.nlm.nih.gov/bioproject/PRJNA953169; BioProject ID: PRJNA953169).

## List of abbreviations

ASD: Autism spectrum disorder
OTUs: Operational taxonomic units
KEGG: Kyoto Encyclopedia of Genes and Genomes
COG: Clusters of Orthologous Groups
ALA: Alpha-linolenic acid
PDD-NOS: Pervasive developmental disorders not otherwise specified
PCoA: Principal coordinate analysis
LEfSe: Linear discriminant analysis effect size
LDA: Linear discriminant analysis
