# Supplementary figures and images for: Gut microbiota signature in children with autism spectrum disorder who suffered from chronic gastrointestinal symptoms
Source: BMC Pediatr. 2023 Sep 20;23:476. doi: 10.1186/s12887-023-04292-8 (PMC10510216; doi:10.1186/s12887-023-04292-8)

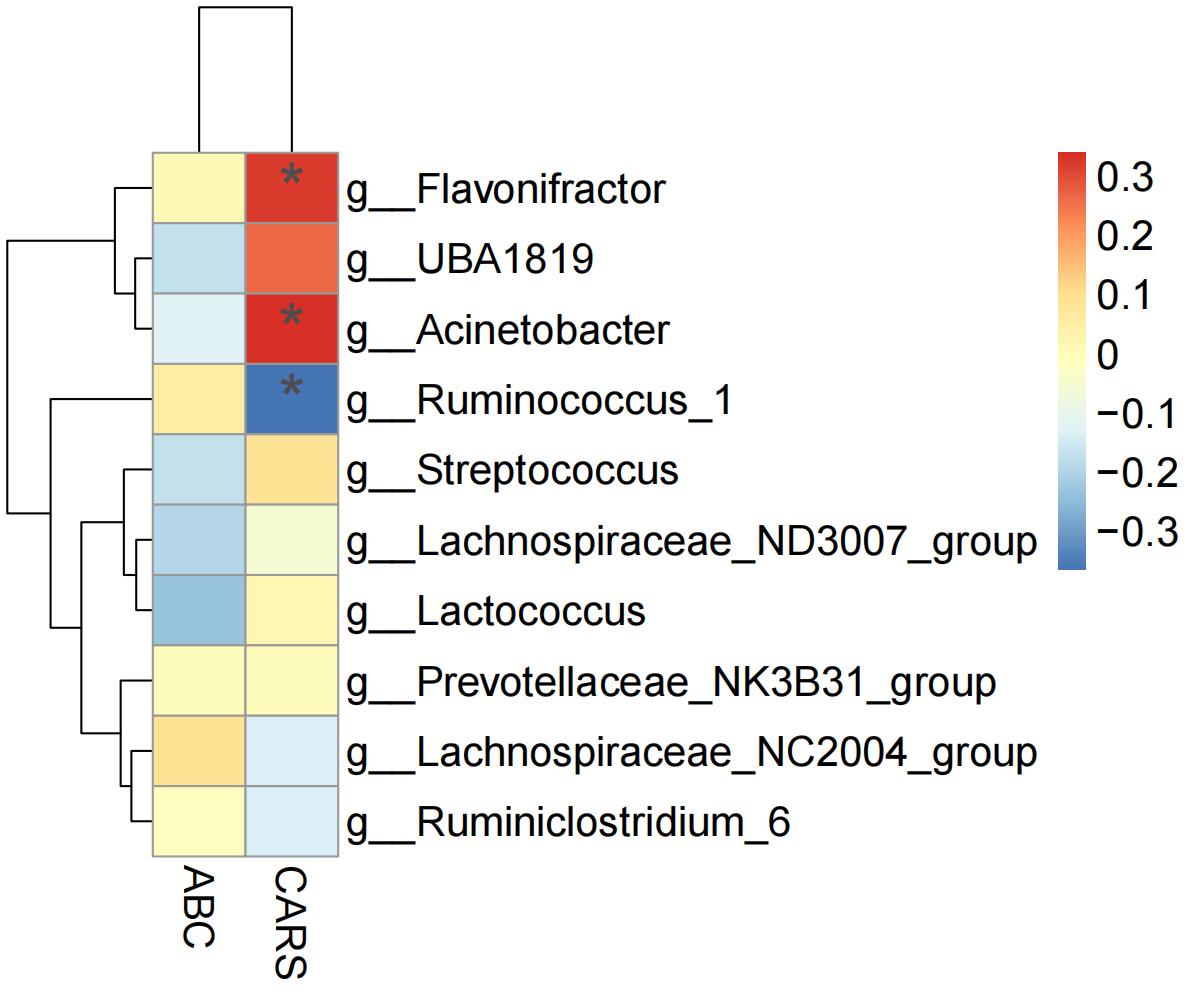

Supplement: Supplementary file 1 — Supplementary Material 1 [file 12887_2023_4292_MOESM1_ESM.jpg]
