# Supplementary material for: Gut microbiota signature in children with autism spectrum disorder who suffered from chronic gastrointestinal symptoms
Source: BMC Pediatr. 2023 Sep 20;23:476. doi: 10.1186/s12887-023-04292-8 (PMC10510216; doi:10.1186/s12887-023-04292-8)
Supplement: Supplementary file 2 — Supplementary Material 2 [file 12887_2023_4292_MOESM2_ESM.docx]

**Supplementary figure legends**

**Figure S1** Relationship between differentially abundant taxa and clinical indices (ABC and CARS) in ASD group by Spearman’s correlation analysis. ABC, Autism Behavior Checklist; CARS, Childhood Autism Rating Scale.
