# Supplementary material for: Gut microbiota signature in children with autism spectrum disorder who suffered from chronic gastrointestinal symptoms
Source: BMC Pediatr. 2023 Sep 20;23:476. doi: 10.1186/s12887-023-04292-8 (PMC10510216; doi:10.1186/s12887-023-04292-8)
Supplement: Supplementary file 10 — Supplementary Material 10 [file 12887_2023_4292_MOESM10_ESM.docx]

Table S8. The fold change of the differential genera in ASD vs control groups

| **Genera** | **ASD** | **Control** | **FC(ASD/Con)** |
| --- | --- | --- | --- |
| *g_Acinetobacter* | 0.0006 | 0.0106 | 0.0606 |
| *g_Flavonifractor* | 0.1176 | 0.2381 | 0.4940 |
| *g_Lachnospiraceae_NC2004_group* | 1.3088 | 0.1679 | 7.7942 |
| *g_Lachnospiraceae_ND3007_group* | 0.1214 | 0.0584 | 2.0791 |
| *g_Lactococcus* | 0.0111 | 0.0010 | 11.2587 |
| *g_Prevotellaceae_NK3B31_group* | 0.0000 | 0.1288 | 0.0000 |
| *g_Ruminiclostridium_6* | 0.1918 | 0.0003 | 727.7500 |
| *g_Ruminococcus_1* | 0.1630 | 0.0527 | 3.0921 |
| *g_Streptococcus* | 2.4507 | 0.5764 | 4.2514 |
| *g_UBA1819* | 0.0176 | 0.1067 | 0.1646 |

ASD, autism spectrum disorder; FC, fold change.
